# *Brucella melitensis* prosthetic joint infection in a traveller returning to the UK from Thailand: Case report and review of the literature

**DOI:** 10.1016/j.tmaid.2016.08.010

**Published:** 2016

**Authors:** Joseph M. Lewis, Jonathan Folb, Sanjay Kalra, S. Bertel Squire, Miriam Taegtmeyer, Nick J. Beeching

**Affiliations:** a Tropical and Infectious Disease Unit, Royal Liverpool University Hospital, Liverpool, UK; b Wellcome Trust Liverpool Glasgow Centre for Global Health Research, Liverpool, UK; c Brucella Reference Unit, Liverpool Clinical Laboratories, Royal Liverpool University Hospital, Liverpool, UK; d Department of Orthopaedics, Royal Liverpool University Hospital, Liverpool, UK; e Department of Clinical Sciences, Liverpool School of Tropical Medicine, Liverpool, UK; f NIHR Health Protection Research Unit in Emerging and Zoonotic Infections, University of Liverpool, L69 7BE, UK

**Keywords:** Brucellosis, Prosthetic joint infection, Travel medicine, Returning traveller

## Abstract

**Background:**

*Brucella* spp. prosthetic joint infections are infrequently reported in the literature, particularly in returning travellers, and optimal treatment is unknown.

**Method:**

We describe a prosthetic joint infection (PJI) caused by *Brucella melitensis* in a traveller returning to the UK from Thailand, which we believe to be the first detailed report of brucellosis in a traveller returning from this area. The 23 patients with *Brucella*-related PJI reported in the literature are summarised, together with our case.

**Results:**

The diagnosis of *Brucella*-related PJI is difficult to make; only 30% of blood cultures and 75% of joint aspiration cultures were positive in the reported cases. Culture of intraoperative samples provides the best diagnostic yield. In the absence of radiological evidence of joint loosening, combination antimicrobial therapy alone may be appropriate treatment in the first instance; this was successful in 6/7 [86%] of patients, though small numbers of patients and the likelihood of reporting bias warrant caution in drawing any firm conclusions about optimal treatment. Aerosolisation of synovial fluid during joint aspiration procedures and nosocomial infection has been described.

**Conclusions:**

*Brucella*-related PJI should be considered in the differential of travellers returning from endemic areas with PJI, including Thailand. Personal protective equipment including fit tested filtering face piece-3 (FFP3) mask or equivalent is recommended for personnel carrying out joint aspiration when brucellosis is suspected. Travellers can reduce the risk of brucellosis by avoiding unpasteurised dairy products and animal contact (particularly on farms and abattoirs) in endemic areas and should be counselled regarding these risks as part of their pre-travel assessment.

## 1 Introduction

Brucellosis is a zoonotic infection transmitted to humans from fluids of infected animals or through consumption of unpasteurised dairy products [1]. It is caused by *Brucella* spp., intracellular Gram-negative coccobacilli. Four species cause most cases of human disease, each with a different animal host reservoir: *Brucella melitensis* (goats, camels) is most common, followed by *Brucella abortus* (cattle), *Brucella suis* (pigs) and *Brucella canis* (dogs). Infections with new species such a *Brucella pinnepedialis* and *Brucella ceti* (marine animals) are occasionally recognized [2]. It can cause an acute febrile illness after a usual incubation period of 1–4 weeks, ranging up to 6 months, or chronic infection, which can be without focus or can affect any organ system. Osteoarticular involvement is the most common focal presentation. Diagnosis is usually based on serology, augmented when possible by culture of *Brucella* organisms from blood, synovial fluid, or bone. Promising molecular methods are in development. Treatment is usually with combination therapy of doxycycline, rifampicin ± an aminoglycoside for 6–12 weeks [1]. Prosthetic joint infections (PJI) caused by *Brucella* spp. are uncommonly reported in the literature. We describe a PJI caused by *B. melitensis* in a traveller returning to the UK from Thailand, the first detailed report of brucellosis in a traveller returning from this area; we also present a review of the 24 reported cases of Brucella-related PJI in the literature.

## 2 Materials and methods

### 2.1 Case report

A 51-year old UK resident attended our clinic on 5 May 2015 with a 21-day history of daily rigors, profuse sweating attacks and high fever. He had returned from Thailand three months earlier. He also had pain and swelling in his left knee, in which he had an uncomplicated total knee replacement 5 years previously for early onset osteoarthritis following trauma. The only abnormalities on examination were fever of 38.3 °C and a small effusion in the symptomatic knee. Blood cultures yielded Gram-negative coccobacilli after 3 days (BioMerieux Bact/ALERT blood culture system), identified as *B. melitensis* by matrix-assisted laser desorption/ionization time-of-flight (MALDI-TOF) mass spectrometry (Bruker microflex LT), but not before two laboratory scientists had been exposed to open bacterial culture plates. The organism was confirmed as *B. melitensis* biotype 3 in the Veterinary Investigation Centre in Weybridge. Standard agglutination tests for brucellosis were suggestive of chronic infection, with IgG titres of >1:2560 and IgM 1:80.

Aspiration of the knee was carried out by the orthopaedic team, equipped with personal protective equipment [PPE] consisting of gown, gloves, apron, visor and filtering face piece-3 [FFP3] respirator. Cloudy fluid was aspirated; this contained over 6000 lymphocytes/mm^3^ and cultured *B. melitensis* after 7 days. The patient commenced doxycycline and rifampicin 600 mg daily for 6 months, together with parenteral gentamicin 5 mg/kg/day for the first 14 days, with resolution of his symptoms and preservation of his implant without revision surgery. Twelve months later he has fully recovered with no signs of loosening of the joint prosthesis on plain x-rays. The exposed laboratory personnel were given doxycycline 100 mg twice daily for 21 days as postexposure prophylaxis according to UK guidelines [3].

The patient made frequent visits to Thailand where he had most recently stayed with a friend on his farm in Nakom Pathom province from 11 December 2014 to 8 January 2015. During that time, he helped deliver several parturient goats and handled newly born kids and other products of conception with his bare hands. He had not consumed unpasteurised dairy products and had no contact with cattle or buffaloes. Two farm workers had contemporaneous fevers, only recognised to be due to brucellosis and treated appropriately after our patient was diagnosed.

### 2.2 Literature review

PubMed and Scopus databases were searched using the search string (((((((prosth*) *OR replacement*)) *OR arthroplasty*)) *AND* (((*knee*) *OR hip*) *OR joint*))) AND brucell*. Studies were reviewed and data extracted by one author (JL), with no restriction on date or language. Prosthetic joint brucellosis was defined as either a) *Brucella* spp. recovered from prosthetic joint synovial fluid culture OR b) signs and symptoms consistent with PJI AND *Brucella* spp. recovered from blood OR positive serology (standard agglutination test [SAT] titre > 1:160 OR fourfold rise in titre between acute and convalescent samples).

## 3 Results and discussion

The search returned 48 results in Scopus and 26 in PubMed. After removal of duplicates, 47 remained. 18 reports contained data on 23 patients with 26 *Brucella*-related prosthetic joint infections; only 3 were in returning travellers [4], [5], [6], [7], [8], [9], [10], [11], [12], [13], [14], [15], [16], [17], [18], [19], [20], [21]. Table 1 summarises all 24 patients including: gender, country of exposure, type of implant and time to symptom onset. In all cases *Brucella* spp. were recovered from blood, synovial fluid or operative tissue sample. No diagnoses were made using serology alone.

It is possible to draw several conclusions from these cases; *Brucella-*related PJI is a late complication of joint arthroplasty, with a median onset of 36 months after the procedure. The diagnosis can be difficult to make: only 30% (3/10) of reported blood cultures and 75% (9/12) of reported joint aspiration samples cultured *Brucella* organisms. Culture of intra-operative tissue samples probably provides the best yield and confirmed the diagnosis in 15/24 cases; in these 15 cases joint aspiration was either not carried out (12/15) or was culture-negative (3/15). In the absence of radiological evidence of implant loosening, medical management with antibiotics alone appears to be effective in the first instance; of 24 patients with 27 infected prosthetic joints, 7 patients (with 9 infected prosthetic joints) had radiologically well-seated implants with no abscess or draining sinus. These patients underwent antibiotic treatment alone for between 6 and 52 weeks, with cure in 6/7 patients (8/9 joints) and failure of medical therapy necessitating surgery in only one patient (one joint). One patient with an infected joint that was radiologically well seated had a draining sinus, but was successfully treated with debridement and adjuvant antibiotics without explant of the prosthesis. However, caution must be exercised in drawing firm conclusions on optimal treatment from these data, given the small numbers and the likelihood of selection bias inherent in case reports.

Sixteen patients (with 17 infected joints) had features of loosening on imaging; these all underwent either 1- or 2-stage revision of their prosthesis alongside antibiotic therapy, all with favourable outcome. One patient was lost to follow up. Follow up was for a median of 24 months.

These cases also provide some guidance on appropriate infection control measures when considering a diagnosis of *Brucella-*related PJI. Infection of laboratory staff by exposure to *Brucella* spp. is well recognised. Procedures that generate aerosolized bacteria provide the highest risk of exposure [22]. Synovial fluid from *Brucella*-infected joints is likely to have a lower bacillary load than culture bottles or plates and therefore exposure to synovial fluid during joint aspiration or joint revision surgery probably represents a lower risk exposure. Nevertheless, a case of transmission during joint aspiration has been described, to a radiology technician who assisted with injecting synovial fluid from a *Brucella*-infected joint from a syringe into a sample container [20]. Neither UK [3] nor US guidelines [23] provide recommendations for risk assessment of potential *Brucella* exposure outside the laboratory, or recommendations for PPE while performing joint aspiration or surgery. We recommend that healthcare workers undertaking aspiration of or surgery on joints in which *Brucella* infection is suspected or confirmed are outfitted with PPE including gown, visor and fit-tested FFP3 respirator or equivalent.

Brucellosis is not a diagnosis that would usually be considered in a traveller returning from Thailand [24]. Two cases acquired in Thailand have been mentioned in passing in reviews of children [25] and adult [26] travellers returning to North America and Europe respectively. Foci in China, Mongolia and Central Eurasia are well recognised but the range of other countries newly affected by brucellosis continues to expand [2], [27], [28], [29], [30]. Human infections are under-reported compared to the patchy knowledge of its increasing incidence in livestock in South Asia [31]. A boy acquired brucellosis from raw goat's milk in Penang, Malaysia in 2010 and a German visitor acquired brucellosis in Myanmar from drinking lassi [32]. An outbreak of caprine and human brucellosis in Ratchaburi Province in Thailand was investigated in 2003 [33] and there have been sporadic case reports and more recent reviews of emerging brucellosis endemicity in Thailand over the past decade [34], [35], [36]. As demonstrated by our patient, the highest risk to humans in Thailand is exposure to parturient goats (*B. melitensis*) but there is a separate risk of *B. abortus* transmission from buffaloes. Diagnosis of illness in travellers can highlight the presence of locally unrecognised infections, as shown by this patient and his contacts.

## 4 Conclusion

In conclusion, we report the first detailed case report of brucellosis in a traveller returning from Thailand. Clinicians should consider brucellosis as well as the more commonly encountered causes of fever in returnees from this area. Brucellosis should be included in the list of possible causes of an infected prosthetic joint in patients who have an appropriate epidemiological risk and PPE, including fit-tested masks, should be used by operators undertaking joint aspiration or surgery in such cases. Though the small number of cases identified in this review warrants caution about drawing any firm conclusions regarding optimal treatment, in the absence of implant loosening, treatment with antibiotics may be appropriate in the first instance. There are no specific strategies for avoidance of *Brucella* spp. PJI beyond those needed by all travellers to prevent brucellosis. These include the avoidance of unpasteurised dairy products (including lassi and buffalo milk or cheese) and animal contact (particularly in farms or abattoirs) in endemic areas. Travellers (with or without prosthetic joints) should be made aware of these risks as part of their standard pre-travel assessment.

## Conflict of interest

Nil.

## Funding

NJB is partially supported by the National Institute for Health Research Health Protection Research Unit (NIHR HPRU) in Emerging and Zoonotic Infections, a partnership between the University of Liverpool and Public Health England, in collaboration with the Liverpool School of Tropical Medicine. NJB is based at the Liverpool School of Tropical Medicine. The views expressed are those of the authors and not necessarily those of the NHS, the NIHR, the Department of Health or Public Health England. JML is supported by the Wellcome Trust as a clinical PhD fellow (grant number 109105/Z/15/Z).

## Figures and Tables

**Table 1:** Summary of 24 patients with *Brucella* spp. prosthetic joint infection.

Reference	Age	Sex	Country of exposure	Traveller	Occupation	Prosthetic implant	Time since implantation (months)	Brucella SAT titre	Radiographic changes	Blood cultures positive	Joint aspirate culture positive	Species	Antibiotics used	Antibiotic course length (weeks)	Surgical management	Follow up (months)	Outcome
Jones et al., 1983 [4]	54	M	USA	No	Dairy farmer	R THR	6	640	No loosening	No	No	*B. abortus*	Tetracycline 500 mg QID Streptomycin 500 mg BID	6 – failed therapy; followed by 52 weeks; Streptomycin first 6 only	One stage revision once medical treatment failed	24	Asymptomatic
Agarwal et al., 1991 [5]	24	F	Saudi Arabia	No	NR	Bilateral TKR	2	2560	No loosening	No	Yes	*B. melitensis*	Rifampicin 300 mg BID Co-trimoxazole 980 mg BID	76	None	19	Pain free, flexion 0–90
Orti et al., 1997 [6]	60	M	Spain	No	“Works with goats”	R TKR	14	160	No oosening	No	Yes	*B. melitensis*	Doxycycline 100 mg BID Rifampicin 900 mg QD Streptomycin 1 g QD	6 Streptomycin first 3 only	None	8	Symptom free
Navarro et al., 1997 [7]	54	M	Spain	No	Shepherd	L internal fixation of femur	324	160	Loosening	No	NR	*B. melitensis*	Doxycycline 100 mg BID Gentamicin 240 mg QD	34 Gentamicin first 1 only	Removal of implant and debridement	18	Asymptomatic
Malizos et al., 1997 [8]	74	M	Greece	No	Shepherd	Bilateral TKR	5	160	No loosening	Yes	Yes	*B. melitensis*	Doxycycline Streptomycin Co-trimoxazole	20 Streptomycin first 3 only	None	24	Asymptomatic
Ortega et al., 2002 [9]	63		Spain	No	Cattle owner	R THR	60	NR	Loosening	No	NR	*B. melitensis*	Doxycycline 100 mg BID Rifampicin 900 mg QD Streptomycin 1 g QD	12 Streptomycin first 3 only	Two-stage revision	6	“Satisfactory”
Weil et al., 2003 [10]	38	M	Israel	No	Artist	L THR	48	1600	Loosening	NR	No	*B. melitensis*	Doxycycline 200 mg QD Rifampicin 600 mg QD	12 6 prior to surgery, 6 after	Two-stage revision	12	Asymptomatic
Weil et al., 2003 [10]	61	M	Israel	No	Retired	R TKR	60	1600	Loosening	NR	No	*B. melitensis*	Doxycycline 200 mg QD Rifampicin 600 mg QD	12 6 prior to surgery, 6 after	Two-stage revision	12	Free of joint pain
Weil et al., 2003 [10]	67	M	Israel	No	Retired	L TKR	168	1600	Loosening	NR	Yes	*B. melitensis*	Doxycycline 200 mg QD Rifampicin 600 mg QD	12 6 prior to surgery, 6 after	Two-stage revision	12	Free of joint pain
Kasim et al., 2004 [11]	47	F	Lebanon	No	NR	L THR	168	640	Loosening	NR	NR	*Brucella* spp.	Doxycycline 100 mg BID Rifampicin 600 mg QD	20	One-stage revision	48	Symptom free, negative *Brucella* titres
Cairo et al., 2006 [12]	50	M	Spain	No	NR	L THR	0	320	No loosening	Yes	NR	*B. melitensis*	Doxycycline 100 mg BID Streptomycin 1 g QD	104 Streptomycin first 2 only	None	60	Well, negative *Brucella* titres
Cairo et al., 2006 [12]	71	M	Spain	No	Farmer	R THR	36	NR	Loosening	No	NR	*B. melitensis*	Doxycycline 100 mg BID Rifampicin 600 mg QD Streptomycin 750 mg QD	24 Streptomycin first week only	Initially one stage revision (infection not suspected); later revision THR after failure of medical therapy	36	Well, negative *Brucella* titres
Cairo et al., 2006 [12]	74	F	Spain	No	NR	L tibial plate	180	80	NR	NR	NR	*B. melitensis*	Doxycycline 100 mg BID Rifampicin 300 mg TID Streptomycin 1 g QD	32 Doxycycline/streptomycin first week Doxycycline/rifampicin for remainder	Initially bone graft and medical therapy – failed – then two stage revision	36	Satisfactory range of movement 0–100° knee
Ruiz-Iban et al., 2006 [13]	66	F	Spain	No	Housewife	THR	36	NR	Loosening	NR	Yes	*B. abortus*	Doxycycline 200 mg QD Rifampicin 900 mg QD	6	Two-stage revision	66	Asymptomatic
Ruiz-Iban et al., 2006 [13]	71	M	Spain	No	Agricultural worker	THR	28	640	No loosening	NR	No	*B. melitensis*	Doxycycline 200 mg QD Rifampicin 900 mg QD Streptomycin 200 mg QD	24 Streptomycin first 6 only	Debridement	60	Asymptomatic
Marbach et al., 2007 [14]	67	NR	Sicily	Yes	NR	Bilateral TKR	48	NR	Loosening	NR	NR	*Brucella* spp.	Doxycycline 100 mg BID Rifampicin 450 mg BID	12	Two-stage revision	15	Good range of movement
Tena et al., 2007 [15]	56	M	Spain	No	Farmer	L THR	60	80	Loosening	No	Yes	*B. melitensis*	Doxycycline 100 mg BID Rifampicin 900 mg QD Streptomycin 1 g QD	8 Doxycycline/streptomycin first 2 weeks Doxycycline/rifampicin for remainder	Two-stage revision	60	Asymptomatic, good joint function
Tassinari et al., 2008 [16]	68	M	Italy	No	NR	R TKR	24	800	No loosening	NR	Yes	*B. melitensis*	Doxycycline 100 mg BID Rifampicin 250 mg QD	8	None	12	Pain disappeared, no radiographic changes
Dauty et al., 2009 [17]	65	F	Portugal	Yes	NR	Bilateral TKR	NR	NR	Loosening	NR	NR	*B. melitensis*	Doxycycline 200 mg QD Rifampicin 900 mg QD	12	Two-stage revision	120	Pain free, walking distance > 1 km
Erdogan et al., 2010 [18]	63	F	Turkey	No	NR	R TKR	24	160	NR	NR	NR	*B. melitensis*	Doxycycline 200 mg QD Rifampicin 600 mg QD	20 Initially 6 weeks, followed by revision TKR, then 16 weeks	One-stage revision	36	Free of joint pain, negative serology
Nichols et al., 2014 [19]	67	F	Mexico	No	NR	THR	24	NR	Loosening	NR	NR	*B. abortus*	Doxycycline Rifampicin	12	Two-stage revision		No evidence of infection recurrence
Lowe et al., 2015 [20]	NR	NR	India	Yes	NR	THR	NR	NR	NR	NR	Yes	*B. melitensis*	None – lost to follow up	N/a	None	0	Unknown
Carothers et al., 2015 [21]	67	F	USA or Mexico	No	NR	R THR	24	NR	Loosening	NR	NR	*B. abortus*	Doxycycline 100 mg BID Rifampicin 300 mg BID	20	Two-stage revision	24	Well, no evidence of infection
Present case	51	M	Thailand	Yes	Company director	L TKR	60	>2560	No loosening	Yes	Yes	*B. melitensis*	Doxycycline 200 mg QD Rifampicin 600 mg QD Gentamicin 400 mg QD	24 Gentamicin first 2 weeks only	None	12	Well, pain free, fully mobile, no radiographic changes

M = male, F = female, L = left, R = right, NR = not reported, SAT = Standard agglutination test, QD = quaque die [once daily], BID = bis in die [twice daily], TID = ter in die [thrice daily], QID = quater in die [four times daily], THR = total hip replacement, TKR = total knee replacement. Where dose and/or dosing interval are given in original report, they are reproduced here.
